# Psychological predictors of medical residents’ perspectives on shared decision-making with patients: a cross-sectional study

**DOI:** 10.1186/s12909-020-02179-1

**Published:** 2020-08-05

**Authors:** Farzan Kheirkhah, Reza Mousavi Larijani, Mahbobeh Faramarzi, Mohamahd Hadi Yadollahpour, Soraya Khafri

**Affiliations:** 1 Infertility and Reproductive Health Research Center, Health Research Institute, Babol University of Medical Sciences, Babol, Iran; 2 Student Research Committee, Babol University of Medical Sciences, Babol, Iran; 3 Social Determinants of Health Research Center, Health Research Institute, Babol University of Medical Sciences, Babol, Iran

**Keywords:** Decision making, Medical residents, Personality, Mental health, Openness to experience

## Abstract

**Background:**

Shared Decision Making (SDM) is as ideal model for resident-patient relationship which may improve medical outcomes. Nevertheless, predictive psychological factors influencing residents’ perspective regarding SDM are unclear. The current study investigated the relationship between two psychological factors, mental health and personality traits, and residents’ views toward SDM.

**Method:**

In a cross-sectional study, 168 medical residents of the Babol University of Medical Sciences studying in 13 field specialties were recruited. The residents completed three questionnaires including Shared Decision-Making Questionnaire (SDM-Q-Doc, physician version), General Health Questionnaire (GHQ-12), and Big Five Personality (NEO-FFI).

**Results:**

Residents had an overall agreement of about 88% regarding SDM with patients. There was no significant difference between male and female residents in terms of the degree of agreement for SDM. Concerning SDM, there was no significant relationship either between residents’ views and neuroticism, extraversion, agreeableness, and conscientiousness. In multivariate regression, mental health did not predict the SDM, but openness to experience negatively predicted residents’ views concerning SDM (β = − 0.388, *p* < 0.001).

**Conclusion:**

The residents’ personality trait of openness to experience was a significant negative predictor of SDM with patients. A better understanding of how psychological factors relate to residents’ perspectives may help clinicians properly discuss the treatment options with the patient thereby encouraging them for SDM or to consider their own preferences.

## Background

Shared Decision-Making (SDM) is an important part of patient-focused care. This process refers to a set of evidence-based communication and practice skills between the patient and the physician which clarifies the patient’s expectations and any maladaptation, while also discussing the best evidence available for the benefits and disadvantages of treatment [1, 2]. SDM has been promoted in many health care systems and has become very important internationally [3, 4]. One of the reasons for this change is the increase in the knowledge of patients about their illnesses and treatments through the media, the increasing number of treatment options available, and the preference of both patients and physicians for greater involvement of the patient [4, 5].

SDM depends on establishing a good therapeutic alliance between physicians and patients for sharing information and supporting patients to express their views during the decision-making process. Some factors such as, individual personality traits and learned experiences/ beliefs/behaviors, and personal beliefs may influence communication style, and approach to SDM. An appropriate model to achieve SDM consists of three parts: the right to choose the option, describing the options of the treatment, and assisting the patient in the decision-making [6]. SDM helps patients and physician agree on long-term care decisions [4]. It is also associated with individual autonomy, control, and feelings of merit [7]. Greater participation of patients in the treatment can be considered a moderator for patient satisfaction [8].

While there is evidence that employing SDM may improve medical outcomes [9–11], the integration of these principles into clinical practice has been uncommon [12, 13]. There are several challenges to the successful adoption of SDM including system-related issues, patient-related issues, and practitioner-related issues [14]. Little research exists on the psychological factors influencing SDM in practical settings [15]. A study revealed that the common important factors related to implementation of SDM in clinical setting included the quality of professional-patient relationship, knowledge about treatment options, information provision about treatment options as well as potential harms and benefits, and professional explanations about the course of care [16]. A cohort study explored the relationships between five factors of personality and four preference types of patient decision-making styles. The results emphasized that increased conscientiousness and openness to experience and decreased agreeableness and neuroticism corresponded to preferring the most active SDM style compared with the least active [17]. Also, few studies explored the factors affecting residents’ perspectives toward SDM [18].

However, the relationship between psychological factors and residents’ views toward SDM has remained unexplored. According to Bowlby’ attachment theory (1973), an individual’s initial attachment is established early in the development with primary caregivers provides a cognitive framework for later social relationships [19]. The evidence supports that attachment styles are shaped by the first relationships have an influence on individual’s personality development and interpersonal relationships [20–22]. The attachment styles shaped by early childhood experiences can provide important contributions to understanding individuals’ decision making styles. Also, attachment styles explain decision making styles and personality traits. The evidence confirmed that attachment styles significantly predict both the decision making styles and five-factor personality traits [23, 24]. To address the gaps of the knowledge about SDM in residents, this study aimed to describe the residents’ prescriptive about implementing SDM in clinical settings along with some related psychological factors predicting the residents’ views toward SDM. To the best of our knowledge, this is the first study examining the relationship between two psychological factors including mental health and personality traits and residents’ views toward SDM. The aims of the study were to test: 1) the residents’ perspective on SDM in clinical settings 2) gender differences regarding to medical residents’ perspectives on SDM with patients, mental health status of residents, and personality traits of residents 3) the roles of five-factor personality traits and mental health as predictive factors of medical residents’ perspectives on SDM with patients.

## Methods

An analytical cross-sectional study was conducted on residents at Babol University of Medical Sciences from August to October 2018. This study was approved by Ethics Committee of Babol University of Medical Sciences (IR.MUBABOL.HRI.REC.1397.056). The study was conducted in compliance with the requirements of the Declaration of Helsinki. Participants’ names were not recorded to assure confidentiality. Also, the participants had right to make informed decision regarding participation in the research. All medical resident participants gave written informed consent at the beginning of the study.

Census sampling was used in this study. All of the 234 medical residents of the Medical School of Babol University of Medical Sciences in 13 field specialties were invited to participate in the study. The specialties included Pediatrics, psychiatry, radiology, internal medicine, orthopedic surgery, obstetrics and gynecology, pathology, anesthesiology, infectious disease, surgery, radiology oncology, cardiology, and urology. One of the research team members visited the residents individually and explained the aims of the study and invited them to attend the study. If the resident was willing to participate in the study, the research team member delivered the questionnaires to her/him with an unnamed envelope. The envelope contained questions about demographic characteristics (age, sex, field of study), along with the questionnaires. A nurse outside the research team was responsible for collecting the completed questionnaires. After 1 week, the nurse asked the residents if they had completed the questionnaires via an SMS message. If the questionnaires were completed, the nurse collected the completed questionnaires in a sealed envelope and returned to the research team. A total of 168 residents completed all of the questionnaires. However, with total number observation 168 residents, the power test of linear regression analysis was 0.761.

We conducted study to address three hypothesis: 1) to describe the the residents’ perspective on SDM in clinical settings with Shared Decision-Making Questionnaire (SDM-Q-Doc, physician version, the mental health of residents with General Health Questionnaire (GHQ-12), and presonality traites with Big Five Personality (NEO-FFI) 2) to test whether was gender differences regarding to medical residents’ perspectives on SDM with patients, mental health status of residents, and personality traits of residents 3) to test whether five-factor personality traits and mental health were as predictive factors of medical residents’ perspectives on SDM with patients.

### Questionnaires

#### Shared decision-making questionnaire (SDM-Q-doc, physician version)

The SDM-Q-Doc is a well-accepted and reliable instrument for assessing the physicians’ perspective on SDM in clinical settings [25]. The questionnaire contains 9 questions where students rate their views on the extent to which they agree on a collaborative clinical decision with the patient from 0 (completely disagree) to 5 (fully agree), with the scores ranging from 0 to 45. Higher scores imply a more positive and consistent view of students towards making a collaborative clinical decision with the patient. The validity of the Iranian version of the SDM-Q-Doc questionnaire was confirmed by Ebrahimi et al. [26].

#### General health questionnaire (**GHQ**-**12**)

To assess the mental health of the residents, GHQ with 12 questions was used. GHQ-12 is a valid scale to assess the psychological problems in clinical settings [27]. Each item of the GHQ-12 is rated on a 4-point scale. We used a bimodal scoring method; “less than usual,” “no more than usual,” with both being worth 0 points, plus “rather more than usual,” or “much more than usual” with each score representing 1 point. This scale determines the mental health on a scale of 0–12 with a higher score revealing a worse condition. The validity of the Persian version of this questionnaire had been evaluated in a study by Tagharrobi et al. [28]. That study reported the cutoff GHQ-12 > 3.7 for mental distress.

#### Big five personality (NEO-FFI)

This scale measures the traits defined by the Five Factor Theory of Personality adapted from Goldberg (1992). It consists of 60 items with 5-point rating (1 = strongly disagree to 5 = strongly agree). The five factors include neuroticism (instability, the polar opposite of emotional stability), extraversion (talkativeness, assertiveness, and energy), openness (originality, curiosity, and ingenuity), agreeableness (good-naturedness, cooperativeness, and trust), and conscientiousness (orderliness, responsibility, and dependability) [29]. This study used the valid Persian NEO-FFI [30].

### Statistical analysis

The descriptive statistics reported included means and frequencies. T student tests were used to compare the mean scores of SDM, mental health, and personality traits between male and female residents. Pearson correlation coefficient was used to test possible significant relationships between SDM scores of the residents and mental health, as well as personality traits. Finally, a multiple hierarchical regression model (method enter) was used to find the predictors of residents’ perspectives regarding SDM. The variables included in the model were those showing a significant correlation with SDM scores in a previous bivariate association. The demographic variable of age was considered as an adjusted variable. Data analysis was carried out using SPSS version 22. The level of significance in all analyses was considered as *p* < 0.05.

## Results

Our response rate was 168 of 234 residents (72%). Table 1 presents the characteristics of respondents, with about 60% male responsiveness. In terms of age, 48.5, 43.5, and 7% of residents had age ranges of 24–30 years, 31–35 years, and 36–50 years, respectively. The mean value of residents’ perspectives towards SDM was 29.00 ± 3.8.

**Table 1:** Demographic characteristics of population study

Variables	
Gender (n,%)	
Male	100 (59.2)
Female	69 (40.8)
Age (Mean, SD)	31.49 ± 3.72
**Field of specialty**	**N %**
Orthopedic surgery	16 (9.7)
Psychiatry	9 (5.5)
Pathology	7 (4.3)
Cardiology	11 (6.7)
Radiology Oncology	4 (2.4)
Obstetrics and Gynecology	17 (10.4)
Internal medicine	23 (14.1)
Surgery	17 (10.4)
Pediatrics	21 (12.8)
Anesthesiology	14 (8.5)
Infectious disease	8 (4.8)
Radiology	13 (7.9)
Urology	4 (2.4)

Nine aspects of SDM-Q-Doc were investigated in terms of residents’ perspective regarding SDM with patients. As shown in Table 2, the residents showed an overall agreement of about 88% for making shared decisions with patients. The analysis also revealed that the residents had moderate agreement (somewhat agree) with the 9-item SDM-Q-Doc scale (ranging from 42 to 79%). The highest disagreement of residents for making shared decisions was related to item 6 (“I asked my patient which treatment option he/she would prefer”) with 20.7% disagreement, and item 7 (“My patient and I thoroughly weighed the different treatment options”) with 44.2% disagreement.

**Table 2:** Frequency and percentile of medical residents’ perspectives regarding to the 9-item Shared Decision Making Questionnaire (SDM-Q-Doc, physician version)

	Completely disagree		Strongly disagree		Somewhat disagree		Somewhat agree		Strongly agree		Completely agree		***P***-value
	Male N (%)	Female N (%)	Male N (%)	Female N (%)	Male N (%)	Female N (%)	Male N (%)	Female N (%)	Male N (%)	Female N (%)	Male N (%)	Female N (%)	
1. I made clear to my patient that a decision needs to be made	0 (0)	0 (0)	1 (1)	0 (0)	5 (5)	6 (8.7)	70 (70)	45 (65.2)	20 (20)	16 (23.2)	4 (4)	2 (2.9)	0.732
2. I wanted to know exactly from my patient how he/she wants to be involved in making the decision	0 (0)	0 (0)	5 (5)	2 (2.9)	66 (66)	44 (63.8)	26 (26)	22 (31.9)	3 (3)	1 (1.4)	0 (0)	0 (0)	0.710
3. I told my patient that there are different options for treating his/her medical condition	0 (0)	0 (0)	0 (0)	0 (0)	7 (7)	5 (7.2)	64 (64)	37 (53.6)	25 (36.2)	27 (27)	2 (2.9)	2 (2.0)	0.510
4. I precisely explained the advantages and disadvantages of the treatment options to my patient	1 (1)	0 (0)	3 (3)	4 (5.8)	10 (10)	5 (7.2)	54 (54)	38 (55.1)	31 (31)	20 (29)	1 (1)	2 (2.9)	0.746
5. I helped my patient understand all the information	2 (2)	2 (2.9)	4 (4)	5 (7.2)	15 (15)	7 (10.1)	46 (46)	32 (46.4)	32 (32)	23 (33.3)	1 (1)	0 (0)	0.791
6. I asked my patient which treatment option he/she prefers	0 (0)	0 (0)	1 (1)	5 (7.2)	42 (42)	27 (39.1)	40 (40)	31 (44.9)	16 (16)	5 (7.2)	1 (1)	1 (1.4)	0.117
7. My patient and I thoroughly weighed the different treatment options	0 (0)	0 (0)	00 (0)	1 (1.4)	15 (15)	10 (14.5)	65 (65)	45 (65.2)	20 (20)	13 (18.8)	0 (0)	0 (0)	0.686
8. My patient and I selected a treatment option together	0 (0)	1 (1.4)	15 (15)	10 (14.5)	65 (65)	45 (65.2)	20 (20)	13 (18.8)	0 (0)	0 (0)	0 (0)	0 (0)	0.686
9. My patient and I reached an agreement on how to proceed	0 (0)	0 (0)	4 (4)	0 (0)	2120))	11 (15.9)	64 (64)	44 (63.8)	12 (12)	14 (20.3)	0 (0)	0 (0)	0.175

Also, Table 2 indicates that there is no significant difference between male and female residents in terms of the degree of agreement on SDM with patients in any of questions of the 9-item SDM-Q-Doc (*P* < 0.05).

Table 3 compares the mean and standard errors of the psychological variables between males and females. According to cutoff GHQ-12 > 3.7, about 28.5% (48 residents) had mental distress. Of 48 residents with psychological distress, 20 persons were women (41.7%) and 28 male (59.3%). There were no differences between female and male residents regarding the mental distress (*P* < 0.05). Also to adherence to, safeguarding participants protocol and support of residents who suffered from mental disorders, we suggested to the residents with GHQ-12 > 3.7 (as suspect to mental disorders) to have consult with a psychiatric. Analysis with *t* student tests suggested that mean values of the shared decision-making, mental health, spiritual health, and all of the five factors of personality traits were not significantly different between male and female residents.

**Table 3:** Mean and standard deviations of shared making decision, mental health, and personality of medical residents

Variables	Men Mean (SD)	Women Mean (SD)	*P*-value
Shared Decision Making	29.37 (4.12)	29.46 (3.90)	0.924
Mental Health	2.39 (2.19)	2.33 (2.37)	0.790
Personality			
Neuroticism	18.82 (3.05)	18.51 (2.76)	0.394
Extraversion	27.43 (1.99)	27.43 (1.69)	0.983
Openness to experience	29.38 (2.94)	29.49 (2.33)	0.751
Agreeableness	24.44 (2.54)	23.92 (2.04)	0.073
Conscientiousness	29.16 (1.75)	29.08 (1.81)	0.694

**Ranges scores:** Shared Decision Making 0–45, Mental Health 0–12, Existential well-being 10–60, Religious well-being 10–60, Total scores of spiritual well-being 20–120, Neuroticism 12–60, Extraversion 12–60, Openness to experience 12–60, Agreeableness 12–60, Conscientiousness 12–16

Table 4 shows the correlation of residents’ views regarding SDM and mental health and all of the five factors of personality traits. The results of Pearson correlation coefficient test revealed a negative significant relationship between residents’ views regarding SDM and mental health of the residents. Also, there was a negative significant relationship between residents’ views regarding SDM and openness to experience. On the other hand, there was no significant relationship between residents’ views regarding SDM and neuroticism, extraversion, agreeableness, as well as conscientiousness.

**Table 4:** Matrix Pearson correlation between medical residents’ perspectives regarding shared decision making with patients and psychological factors

Variables	1	2	3	4	5
1. Shared Decision Making					
2. Neuroticism	0.030				
3. Extraversion	−0.130	0.001			
4. Openness to experience	−0.391**	0.335**	0.371**		
5. Agreeableness	− 0.071	0.197*	−0.135	− 0.274*	
6. Conscientiousness	-o.054	−0.052	0.211*	0.212*	0.092

Significance level: *p < 0.055, **p < 0.01

Table 5 reports the results of multivariate linear regression tests between residents’ views regarding SDM and personality characteristics along with mental health. Two variables that correlated significantly with residents’ views regarding SDM, mental health and openness to experience, were introduced into the regression model. The final analysis showed that only openness to experience negatively predicted the residents’ views towards SDM (β = − 0.388, *p* < 0.001).

**Table 5:** Psychological of predictors of medical residents’ perspectives regarding shared decision making with patients

Variables	Unstandard β (SE)	Standard β	*P* Value
Openness to experience	−0.27 ± 0.1	−0.19	0.013
Mental Health	1.14 ± 0.65	0.14	0.075

## Discussion

The findings of the study suggested that residents had a positive view of making shared decisions with patients. Specifically, 88% of residents agreed with SDM. McKeown et al. reported that first-year residents felt strongly about the need to share information with the patients [31]. A meta-analysis including 43 papers reported that the physicians’ perspective toward SDM was positive in treatment decisions and clinical practice, through the physician support for SDM was considered inadequate [32].

The present study revealed no relationship between male and female residents regarding SDM. In line with this study, Thompson et al. reported that there were no gender differences in preferences for involving patients in medical decision-making [33]. In contrast with this result, some previous studies reported that females have greater preference for SDM with patients compared to men [17, 34].

Our findings suggested that the psychological factors of mental health and all of the five factors of personality traits were not significantly different between male and female residents. Although many previous studies have reported different psychological profiles between male and female medical students [35–37], few studies have examined the interactions between psychological factors and gender of the residents. Cillini et al. (2017) investigated residents’ mental health problems via Patient Health Questionnaire-2 (PHQ-2). In that study, the residents had stress (52%), physical exhaustion (41%), mental exhaustion (35%), and PHQ-2-defined depressive symptoms (11%). Also, the female residents reported more physical exhaustion [38].

On the other hand, one characteristic of the personality, openness to experience, negatively predicted the residents’ views regarding SDM. In agreement with our results, a study reported no significant relationship between the five factors of personality (using the NEO–Five Factor Inventory) and preferences for decision-making in patients [39]. However, the results of this study in terms of the relationship between personality traits and decision-making of the residents are not consistent with the association reported elsewhere. Fluny et al. concluded that lower neuroticism and lower agreeableness were associated with less participation in SDM with patients. In that study, higher conscientiousness and openness to experience scores were observed among practitioners who would prefer to make shared decisions with patients [17].

The negative predictive role of openness to experience in SDM with the patient could be examined based on its components. Openness to experience consists of five dimensions including fantasy, aesthetics, feelings, actions, ideas, and values. Open individuals are not passive recipients of experience; rather, they actively look for new experiences; they are intellectually challenging, and they may explore novelty [40]. Thus, we hypothesize that more open residents would report higher levels of agreeableness and higher SDM with patients. We also present some evidence for confirming this finding which is opposite to our first idea and some previous studies.

First, the personality traits of medical residents may be different from those of the general population. For testing the hypothesis, two tasks were done; literature review and sub-group analysis of the data. The sub-group analysis of the personality traits in our study via ANOVA test revealed that the mean residents’ openness to experience was significantly higher than that of four other traits, i.e. conscientiousness, extraversion, neuroticism, and agreeableness. Also Pearson correlation tests of our data, revealed that the openness to experiences correlated negatively with agreeableness (r = − 0.274, *P* < 0.05). In reviewing the literature, we found that Hojat et al. reported that the internal residents have greater openness to experience, are less eager to face challenges, and are less able to cope with adversity compared with the general population [41]. Also, personality profiles of the residents revealed that residents were in a high category of openness to experience. For residents who had higher openness to experience, the scores of ideas, feeling, and fantasy were significantly higher than for the general population [41]. Another study reported that higher scores of openness to experience were associated with greater risk-taking [42]. Thus, the residents with higher scores of openness but low agreeableness may have low tendency to SDM.

The second hypothesis proposed personality differences between medical residents and various specialists. Notably, medical residents may have some distinct personal qualities among medical students. A study reported that surgery residents had greater conscientiousness scores compared with pediatric residents: greater extraversion scores compared with first-year medical students and lower openness scores compared with first-year medical students [43]. It is likely that the residents of this study, who had different specialties, may have had different psychological mental profiles including personality traits. Probably, this variety of psychological profiles affected the outcome of these findings, the association between the psychological profiles of residents and their perspectives over SDM. Thus, it is suggested that specialist residency be considered as a confounding factor affecting the psychological profiles of the residents regarding SDM with patients such as emotional intelligence, personal, professional and ethical constructs of compassion, and communication style in future research.

The study had several limitations compromising the generalizability of the results. First, the sampling was limited to a faculty of medicine in Iran. Indeed, only a sample of residents might not be a suitable representative of the other residents. Secondly, the cultural competence may improve the ability of physician on SDM with the patient. Hence, the findings may have been influenced by Iranian contextual cultural factors affecting the psychological profiles of the residents regarding SDM with the patients [44]. In future, multinational studies should be designed to test this hypothesis how contextual cultural factors may affect the profiles of the residents regarding SDM with patients. Finally, the residents with 13 specialties were recruited in the study. The various specialties may have affected the results. In future, multicenter and multinational studies with large sample sizes should be designed to investigate how various specialties of residents, intersubjective factors, prevailing cultures in different types of specialism, individual personality traits and learned experiences/ beliefs/behaviours, and personal beliefs may influence communication style, and approach to SDM. In spite of these limitations in mind, our study highlighted a valuable finding in the SDM research, suggesting that psychological factors of residents should be taken into account when assessing the perspectives to SDM with patients.

## Conclusion

In a cross-sectional study on 168 medical residents, we found that the majority of residents agreed with SDM. The mental health of the residents did not predict the SDM with patients. The personality trait of openness to experience in residents was a significantly negative predictor of SDM with patients. The results provide useful insights into interventions in the training of medical residents for improving physicians’ perspectives on SDM. A better understanding of how psychological factors such as personality traits including openness to experience relate to residents’ perspectives on SDM may help clinicians rightfully participate in treatment opinion exchange with patients and respecting their preferences if required. Further research should explore how personality characteristics of the clinicians relate to SDM with patients.

## Data Availability

The datasets used and/or analyzed during the current study are available from the corresponding author on reasonable request.

## Abbreviations

SDM: Shared Decision Making
SDM-Q-Doc: Shared Decision-Making Questionnaire, physician version
GHQ-12: General Health Questionnaire
NEO-FFI: Big Five Personality
